# The Correlation of Prediabetes and Type 2 Diabetes With Adiposity in Adults

**DOI:** 10.3389/fnut.2022.818263

**Published:** 2022-04-11

**Authors:** Juan Sun, Zhen Liu, Zimu Zhang, Ziyang Zeng, Weiming Kang

**Affiliations:** Division of General Surgery, Department of Surgery, Peking Union Medical College Hospital, Peking Union Medical College, Chinese Academy of Medical Sciences, Beijing, China

**Keywords:** NHANES, diabetes, adiposity, glucose, HbA1c

## Abstract

**Background:**

Fat metabolism is associated with prediabetes and type 2 diabetes mellitus (T2DM). The aim of this study was to evaluate the detailed correlation of diabetes status with adiposity among adults.

**Methods:**

Briefly, 28,429 adults aged ≥18 years from both sexes in the National Health and Nutrition Examination Survey (NHANES) 1999–2018 were included in this study. Multivariable linear regression models were used to examine associations of prediabetes and diabetes status, disease duration of T2DM, serum glucose, glycohemoglobin (HbA1c) with total percent fat (TPF), and fat mass distribution.

**Results:**

After adjusting for sociodemographic covariates, health behaviors, hypertension, hypercholesterolemia, there were direct associations of prediabetes and T2DM status with TPF, trunk fat mass, android fat mass, gynoid fat mass and android to gynoid ratio compared with non-diabetes. But the fat mass decreased with the increase of the disease duration in patients with T2DM. Besides, when stratifying by diabetes status, we found direct associations of serum glucose and HbA1c with TPF, trunk fat mass, android fat mass, gynoid fat mass, and android to gynoid ratio in non-diabetic and prediabetic participants. But in patients with T2DM, inverse associations of serum glucose and HbA1c with fat mass were observed.

**Conclusions:**

This study indicated that adults with prediabetes and T2DM had significantly higher TPF, trunk fat mass, android fat mass, gynoid fat mass, and android to gynoid ratio compared with those without diabetes. Moreover, fat mass decreased as the disease duration increased in patients with T2DM. The associations of serum glucose and HbA1c with TPF and fat mass distribution in patients with T2DM were opposite to the relationships observed in non-diabetic and prediabetic participants.

## Introduction

Prediabetes, with blood glucose concentrations higher than normal, but lower than the threshold of diabetes mellitus (DM), is a high-risk state of DM development (1). The National Health and Nutrition Examination Survey (NHANES) in 2005–2008 suggested that 35% of the US adults older than 20 years and 50% of those older than 65 years had prediabetes (2). Statistically, 5–10% of people per year with prediabetes will progress to diabetes (1) and there are more than 425 million diabetics worldwide, which is expected to reach 700 million in 2,045 (3). The causes of this unprecedented rise are the aging population, increasing obesity, physical inactivity, and energy-dense diets (4). Besides, more than 90% of individuals with MD have type 2 diabetes (T2DM) (5), 60% of patients with T2DM are obese (4), and obesity is one of the strongest factors that drive the increase of the incidence of metabolic diseases, including diabetes (6).

Patients with prediabetes or T2DM need to monitor glycohemoglobin (HbA1c) and blood glucose concentration regularly to prevent the development and progression of microvascular and macrovascular complications (4). Compared with body mass index (BMI), total percent fat (TPF) as well as the indexes of fat distribution by region (trunk fat mass, android fat mass, gynoid fat mass and android to gynoid ratio) are more important and accurate indicators in obesity evaluation. Specifically, dual-energy x-ray absorptiometry (DXA) is widely considered a precise and accurate clinical technology for directly measuring fat mass and distribution nowadays (7, 8). In recent years, a significant association between prediabetes or T2DM and adiposity has been established. However, these studies used proxy measures for overall or abdominal obesity such as BMI or waist circumference without taking into account the composition of that mass and the correlation between diabetes status, blood glucose, HbA1c and fat mass measured by DXA in large samples are still limited. Therefore, this study investigated the relationship of diabetes status and disease duration of T2DM with TPF and fat distribution in adults using a nationally representative sample. Furthermore, due to the different blood glucose, HbA1c and adiposity between individuals with and without prediabetes or T2DM, we also evaluated the associations of blood glucose and HbA1c with adiposity in the subgroup stratified by diabetes status.

## Methods

### Data Sources

National Health and Nutrition Examination Survey (NHANES) is a continuous surveillance survey conducted by the Centers for Disease Control and Prevention (CDC) and the National Center for Health Statistics (NCHS) to assess the health and nutritional status of the US population. Data obtained from NHANES can be freely available to researchers worldwide. In our study, we pooled data from 10 2-year cycles of NHANES 1999–2018.

### Study Population

**Figure 1:**
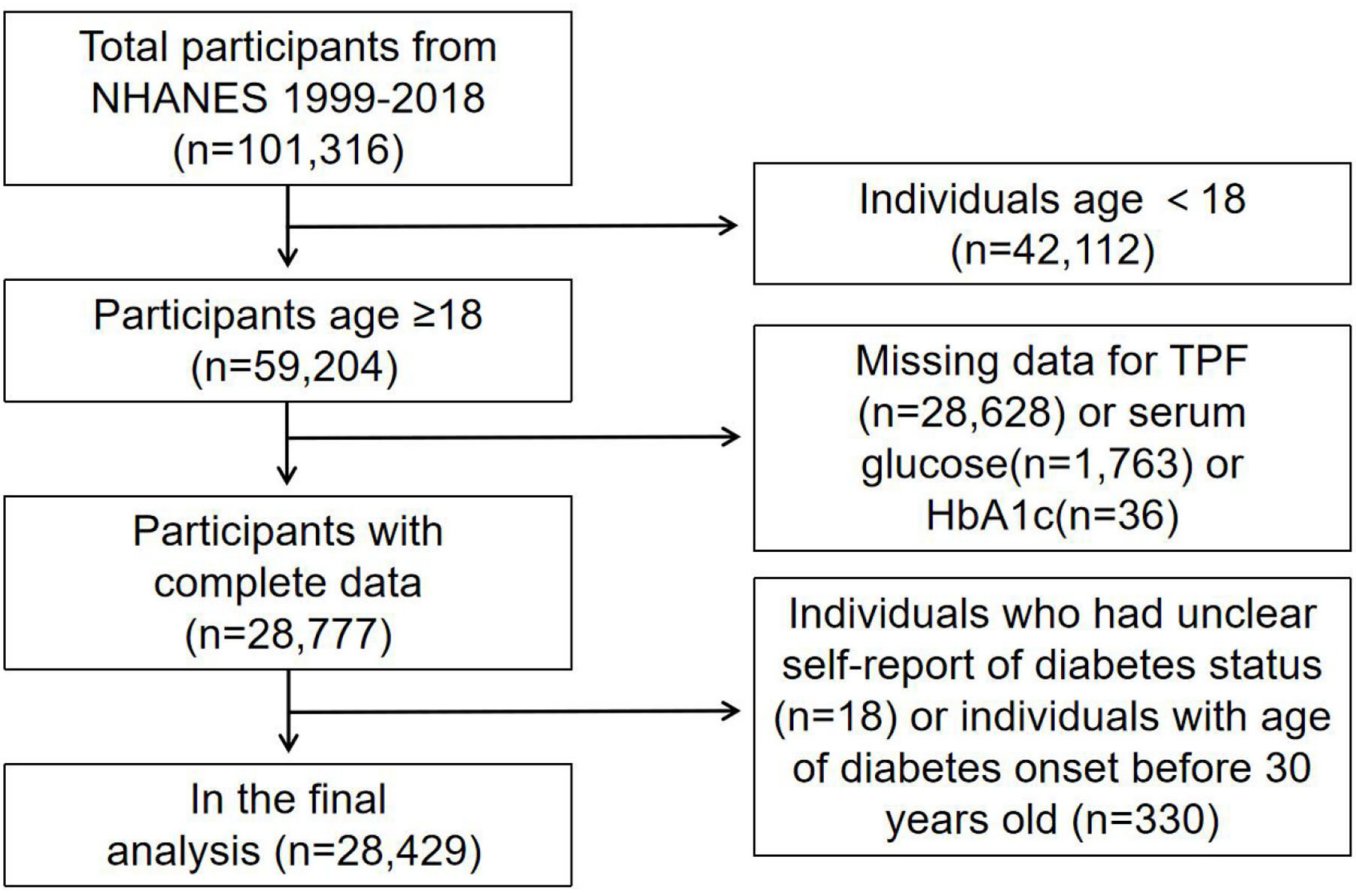
Flow chart of sample selection from the NHANES 1999–2018.

A total of 101,316 participants were enrolled from the NHANES 1999–2018 database. Among the 59,204 adults aged ≥18 years old, we excluded 28628 participants with incomplete TPF data, 1,763 participants with incomplete serum glucose data, 36 participants with incomplete HbA1c data, and 18 participants who had unclear self-report DM status. To minimize the number of participants with T1DM, 330 participants with age of DM onset before the age of 30 were also excluded. Finally, 28,429 participants were analyzed after applying these exclusion criteria (Figure 1).

The National Center for Health Statistics Research Ethics Review Board reviewed and approved NHANES, and all participants signed written consents prior to participating in each year's survey. De-identified data are accessible online.

### Study Variables

The outcomes of our study were TPF, trunk fat mass, android fat mass, gynoid fat mass and android to gynoid ratio, which were measured by well-trained technicians using dual energy X-ray absorptiometry (DXA) QDR-4500 Hologic Scanner (Bedford, MA) of the whole body. TPF was calculated by the ratio of total fat mass to total fat and lean mass and multiplied by 100 to produce a percentage. The regions of fat distribution were defined by the Hologic APEX software. Specifically, the trunk fat mass was defined as total fat mass minus fat in limbs and head. The android area was defined as the lower trunk area bounded by two lines: the pelvic horizontal cut line on its lower side, and a line automatically placed above the pelvic line. The upper gynoid line was placed 1.5 times of the height of android region below the pelvic line and the lower gynoid line was placed such that the distance between the two gynoid lines was twice the height of the android region. DXA scans were not performed for participants with a self-reported use of radiographic contrast material (barium) in the last seven days before scans, weight above 450 pounds or height above 192.5 cm.

Exposures were prediabetes and T2DM status, disease duration in those with T2DM, serum glucose and HbA1c. The T2DM status was defined as self-reported physician-diagnosed diabetes (ever told by a doctor that they had DM) or using measures of HbA1c ≥6.5% in those without a self-reported diagnosis, based on the guideline from the American Diabetes Association (9). The prediabetes status was defined as self-reported physician-diagnosed prediabetes (ever told by a doctor that they had prediabetes or borderline DM) or using measures of HbA1c ≥5.7% and ≤ 6.4% in those without a self-reported diagnosis, based on the guideline from the American Diabetes Association (9). Disease durations in patients with T2DM were defined as the age at screening minus the age when which doctors told the participants they had DM. Serum glucose and Hb1Ac were obtained from the standard biochemistry profile and glycohemoglobin sections in the laboratory data part of NHANES.

Sociodemographic variables mainly included age, sex, race and ratio of family income to poverty threshold. Current insulin use and glucose-lowering medication intake were assessed by questionnaires. Health behaviors variables included smoking status (whether smoked at least 100 cigarettes in life) and vigorous work activity. Health-related variables were hypertension (ever told by a doctor that you have high blood pressure), hypercholesterolemia (ever told by a doctor that you have high cholesterol), BMI and waist circumference (calculated during the study visit). The detailed description of other variables such as total cholesterol, HDL cholesterol, triglyceride, LDL cholesterol as well as blood urea nitrogen, uric acid, creatinine can all be found on the NHANES website (http://www.cdc.gov/nchs/nhanes/).

### Statistical Analyses

The NHANES sample weights were used as recommended by the NCHS. All analyses were performed with package R (http://www.Rproject.org) and Empower Stats (http://www.empowerstats.com), *p*-values <0.05 were considered to indicate statistical significance. The associations between diabetes status, duration of T2DM, serum glucose, HbA1c and TPF or fat distribution were evaluated by multivariable linear regression models. The model was adjusted for age, sex, race, health behaviors (smoking status, vigorous work activity), hypertension, and hypercholesterolemia. Subgroup analyses stratified by sex and diabetes status were also performed.

## Results

The weighted distributions of the characteristics according to diabetes status were shown in Table 1. In 7,802 prediabetic and T2DM patients, 6,674 (85.5%) participants could be diagnosed as obese based on TPF (men 25%, women >35%), and 3,924 (50.3%) could be diagnosed by BMI (≥30 kg/m^2^) (10, 11). Compared with non-diabetic participants, prediabetic, and T2DM participants were older, more had hypertension, hypercholesterolemia, smoked at least 100 cigarettes in life and fewer had vigorous work activity. Besides, prediabetes and T2DM group had higher BMI, waist circumference, total cholesterol, triglyceride, blood urea nitrogen, uric acid, creatinine, and lower ratio of family income to poverty, HDL cholesterol. The mean ± SD of disease duration of diabetes for T2DM group was 6.98 ± 7.97 years and there were 465 patients taking insulin and 1,436 patients taking glucose-lowering medications in all 2,476 patients with T2DM patients. Furthermore, the serum glucose, HbA1c, TPF, trunk fat mass, android fat mass, gynoid fat mass, and android to gynoid ratio in adults with prediabetes or T2DM were all higher than non-diabetic participants.

**Table 1:** Weighted characteristics of study samples based on diabetes status.

	**Non-diabetes** **(*n* = 20,627)**	**Prediabetes** **(*n* = 5,326)**	**Diabetes** **(*n* = 2,476)**	***p-*value**
Sex (%)				0.0155
Males	49.88	48.77	52.76	
Females	50.12	51.23	47.24	
Race (%)				<0.001
Hispanic	14.27	17.66	18.62	
Non-hispanic white	70.44	56.60	56.34	
Non-hispanic black	9.08	17.04	15.85	
Others	6.21	8.69	9.20	
Hypertension (%)				<0.001
Yes	18.17	37.15	58.08	
No	81.83	62.85	41.92	
Hypercholesterolemia (%)				<0.001
Yes	26.11	41.46	58.00	
No	73.89	58.54	42.00	
Vigorous work activity (%)				<0.001
Yes	34.98	24.06	20.58	
No	65.02	75.94	79.42	
Smoking status (%)				<0.001
Yes	44.71	49.00	49.31	
No	55.29	51.00	50.69	
Taking insulin now (*n*)				/
Yes	/	4	465	
No	/	5,199	1,994	
Not available	/	123	17	
Taking pills to lower blood sugar (n)				/
Yes	/	56	1,436	
No	/	946	553	
Not available	/	4,324	487	
Age (years)	39.23 ± 14.39	48.60 ± 13.88	54.45 ± 11.95	<0.001
BMI (kg/m^2^)	27.28 ± 5.86	31.29 ± 7.02	33.24 ± 7.35	<0.001
Ratio of family income to poverty	3.03 ± 1.65	2.89 ± 1.63	2.69 ± 1.61	<0.001
Waist circumference (cm)	93.69 ± 14.77	104.09 ± 15.58	110.97 ± 16.45	<0.001
Total cholesterol (mmol/L)	5.01 ± 1.03	5.31 ± 1.10	5.14 ± 1.30	<0.001
Triglyceride (mmol/L)	1.50 ± 1.31	1.97 ± 1.71	2.55 ± 3.16	<0.001
HDL cholesterol (mmol/L)	1.39 ± 0.41	1.27 ± 0.37	1.20 ± 0.34	<0.001
LDL cholesterol (mmol/L)	2.97 ± 0.88	3.25 ± 0.97	2.93 ± 0.98	<0.001
Blood urea nitrogen (mmol/L)	4.51 ± 1.55	4.79 ± 1.72	5.39 ± 2.56	<0.001
Uric acid (umol/L)	313.83 ± 81.65	335.56 ± 80.70	332.01 ± 91.03	<0.001
Creatinine (mmol/L)	75.56 ± 26.79	76.87 ± 29.90	82.16 ± 58.33	<0.001
Serum glucose (mmol/L)	4.94 ± 0.59	5.52 ± 1.22	8.73 ± 4.25	<0.001
Glycohemoglobin (%)	5.19 ± 0.27	5.84 ± 0.49	7.59 ± 1.90	<0.001
Diabetes duration (years)	/	/	6.98 ± 7.97	/
Total percent fat (%)	32.49 ± 8.66	35.98 ± 8.33	36.85 ± 7.82	<0.001
Trunk fat mass (g)	12489.80 ± 5976.04	16369.64 ± 6781.82	18970.05 ± 7352.68	<0.001
Android fat mass (g)	2154.09 ± 1151.41	2890.19 ± 1325.25	3378.72 ± 1420.56	<0.001
Gynoid fat mass (g)	4382.63 ± 1702.18	5006.58 ± 1950.23	4969.78 ± 1934.28	<0.001
Android to gynoid ratio	0.98 ± 0.20	1.07 ± 0.19	1.13 ± 0.18	<0.001

*Mean ± SD for continuous variables, p-value was calculated by weighted linear regression model. % for categorical variables, p-value was calculated by weighted chi-square test*.

### Associations of Diabetes Status With Adiposity

We found the direct associations of prediabetes and T2DM status with TPF and fat distribution compared with non-diabetes in fully adjusted models (βs in sequence of TPF, trunk fat mass, android fat mass, gynoid fat mass, and android to gynoid ratio for prediabetes were: 2.12/3032.89/609.47/595.10/0.06; for T2DM they were: 2.49/4937.83/ 944.87/582.69/0.08). The results were all statistically significant with *p-*values <0.0001. That is to say, after controlling for potential confounding factors, compared with those without DM, the TPF, trunk fat mass, android fat mass, gynoid fat mass and android to gynoid ratio for prediabetes were 2.12%, 3032.89 g, 609.47 g, 595.10 g, 0.06 higher, and for T2DM were 2.49%, 4937.83 g, 944.87 g, 582.69 g, 0.08 higher. In the subgroup analysis stratified by sex, this direct association existed in both males and females after adjusting for confounders. These results are presented in Table 2.

**Table 2:** Associations of diabetes status with adiposity.

	**β** **(95%** ***CI*****)** ***p-*****value**				
	**Total percent fat (%)**	**Trunk fat mass (g)**	**Android fat mass (g)**	**Gynoid fat mass (g)**	**Android to gynoid ratio**
**Total**
Non-diabetes	Reference	Reference	Reference	Reference	Reference
Prediabetes	2.12 (1.91, 2.34) <0.0001	3032.89 (2809.85, 3255.93) <0.0001	609.47 (558.92, 660.03) <0.0001	595.10 (526.20, 664.01) <0.0001	0.06 (0.05, 0.06) <0.0001
Type 2 diabetes	2.49 (2.17, 2.82) <0.0001	4937.83 (4604.11, 5271.56) <0.0001	944.87 (862.59, 1027.14) <0.0001	582.69 (470.77, 694.62) <0.0001	0.08 (0.07, 0.09) <0.0001
**Males**
Non-diabetes	Reference	Reference	Reference	Reference	Reference
Prediabetes	2.07 (1.78, 2.36) <0.0001	2472.82 (2177.24, 2768.41) <0.0001	491.17 (420.46, 561.88) <0.0001	457.32 (369.05, 545.60) <0.0001	0.04 (0.03, 0.05) <0.0001
Type 2 diabetes	2.85 (2.43, 3.27) <0.0001	4497.33 (4072.30, 4922.36) <0.0001	785.17 (675.45, 894.90) <0.0001	577.37 (440.82, 713.91) <0.0001	0.04 (0.03, 0.06) <0.0001
**Females**
Non-diabetes	Reference	Reference	Reference	Reference	Reference
Prediabetes	2.23 (1.91, 2.55) <0.0001	3619.14 (3289.31, 3948.97) <0.0001	730.95 (659.55, 802.35) <0.0001	743.50 (639.05, 847.95) <0.0001	0.07 (0.06, 0.08) <0.0001
Type 2 diabetes	2.08 (1.59, 2.58) <0.0001	5348.31 (4834.19, 5862.44) <0.0001	1111.89 (989.92, 1233.86) <0.0001	596.94 (418.59, 775.30) <0.0001	0.12 (0.10, 0.13) <0.0001

*Age, sex, race, hypertension, hypercholesterolemia, smoking status, and vigorous work activity were adjusted. Sex was not adjusted for in the subgroup analyses*.

### Associations of Serum Glucose and HbA1c With Adiposity

After adjusting for sociodemographic covariates, health behaviors, hypertension, and hypercholesterolemia, we found direct associations of serum glucose and HbA1c with TPF, trunk fat mass, android fat mass, gynoid fat mass, and android to gynoid ratio. And when stratifying by diabetes status, these direct associations still existed in participants without diabetes and with prediabetes. But in patients with T2DM, the correlation of serum glucose with TPF and trunk fat mass, HbA1c with TPF turned to be inverse (serum glucose and TPF: β = −0.10, 95% *CI*: −0.16~−0.05; serum glucose and trunk fat mass: β = −79.49, 95% *CI*: −147.53~−11.46; HbA1c and TPF: β = −0.13, 95% *CI*: −0.24~−0.01). Other associations were of no statistical significance. The results are shown in Tables 3, 4.

**Table 3:** Associations of serum glucose (mmol/L) with adipoity.

	**β** **(95%** ***CI*****)** ***p-*****value**				
	**Total percent fat (%)**	**Trunk fat mass (g)**	**Android fat mass (g)**	**Gynoid fat mass (g)**	**Android to gynoid ratio**
Total	0.19 (0.13, 0.24) <0.0001	291.60 (231.68, 351.53) <0.0001	73.86 (59.63, 88.08) <0.0001	55.69 (36.28, 75.10) <0.0001	0.01 (0.01, 0.01) <0.0001
Stratified by diabetes status
Non-diabetes	1.32 (1.16, 1.49) <0.0001	1585.22 (1427.90, 1742.55) <0.0001	310.36 (276.47, 344.25) <0.0001	302.24 (254.49, 349.98) <0.0001	0.04 (0.03, 0.04) <0.0001
Prediabetes	0.45 (0.32, 0.58) <0.0001	780.17 (623.60, 936.74) <0.0001	152.58 (117.02, 188.15) <0.0001	100.92 (53.83, 148.01) <0.0001	0.01 (0.01, 0.02) <0.0001
Type 2 diabetes	−0.10 (−0.16, −0.05) 0.0001	−79.49 (−147.53, −11.46) 0.0221	−5.35 (−22.71, 12.01) 0.5461	−19.41 (−41.72, 2.90) 0.0884	0.00 (−0.00, 0.00) 0.5482

*Age, sex, race, hypertension, hypercholesterolemia, smoking status, and vigorous work activity were adjusted*.

**Table 4:** Associations of glycohemoglobin (%) with adiposity.

	**β** **(95%** ***CI*****)** ***p-*****value**				
	**Total percent fat (%)**	**Trunk fat mass (g)**	**Android fat mass (g)**	**Gynoid fat mass (g)**	**Android to gynoid ratio**
Total	0.33 (0.19, 0.46) <0.0001	648.69 (511.22, 786.17) <0.0001	145.88 (112.54, 179.22) <0.0001	107.16 (61.65, 152.68) <0.0001	0.01 (0.01, 0.02) <0.0001
Stratified by diabetes status
Non-diabetes	2.06 (1.69, 2.44) <0.0001	2831.87 (2472.91, 3190.84) <0.0001	535.24 (452.20, 618.27) <0.0001	607.61 (491.33, 723.88) <0.0001	0.04 (0.03, 0.05) <0.0001
Prediabetes	0.82 (0.49, 1.14) <0.0001	1992.63 (1604.47, 2380.79) <0.0001	400.58 (311.38, 489.78) <0.0001	199.11 (80.74, 317.48) 0.0010	0.04 (0.03, 0.05) <0.0001
Type 2 diabetes	−0.13 (−0.24, −0.01) 0.0403	−42.36 (−198.33, 113.60) 0.5945	4.36 (−35.69, 44.41) 0.8312	−34.61 (−86.21, 16.99) 0.1888	0.00 (−0.00, 0.01) 0.2261

*Age, sex, race, hypertension, hypercholesterolemia, smoking status, and vigorous work activity were adjusted*.

### Associations of Disease Duration With Adiposity in T2DM

There were inverse associations of disease duration with TPF and gynoid fat mass, which were statistically significant (β = −0.04, 95% CI: −0.07~−0.01 and β = −18.41, 95% CI: −33.91~−2.91), which could be explained as each unit (1 year) increase in the duration of T2DM, TPF was decreased by 0.04% and gynoid fat mass was decreased by 18.41 g. When stratified by sex, the inverse associations of disease duration with TPF and gynoid fat mass were still statistically significant in females, but not in males. Besides, the inverse associations of disease duration with trunk fat mass, android fat mass and android to gynoid ratio were of no statistical significance either. The results are shown in Table 5.

**Table 5:** Associations of disease duration (years) with adiposity in patients with T2DM.

	**β** **(95%** ***CI*****)** ***p-*****value**				
	**Total percent fat (%)**	**Trunk fat mass (g)**	**Android fat mass (g)**	**Gynoid fat mass (g)**	**Android to gynoid ratio**
Total	−0.04 (−0.07, −0.01) 0.0143	−7.83 (−50.75, 35.09) 0.7208	−7.04 (−19.10, 5.02) 0.2529	−18.41 (−33.91, −2.91) 0.0201	−0.00 (−0.00, 0.00) 0.5460
Males	−0.03 (−0.08, 0.02) 0.2096	−0.90 (−62.59, 60.79) 0.9772	−2.67 (−19.77, 14.42) 0.7593	−8.14 (−28.03, 11.75) 0.4225	−0.00 (−0.00, 0.00) 0.4104
Females	−0.05 (−0.09, −0.00) 0.0370	−11.03 (−70.67, 48.61) 0.7171	−11.86 (−28.84, 5.13) 0.1717	−29.05 (−53.19, −4.92) 0.0186	0.00 (−0.00, 0.00) 0.9603

*Age, sex, race, hypertension, hypercholesterolemia, smoking status, vigorous work activity were adjusted. Sex was not adjusted for in the subgroup analysis*.

## Discussion

The results of our study showed that individuals with prediabetes and T2DM had significantly higher TPF, trunk fat mass, android fat mass, gynoid fat mass, and android to gynoid ratio compared with those without DM. The fat mass decreased as the disease duration increased in patients with T2DM. Moreover, in participants without DM and with prediabetes, serum glucose and HbA1c were directly associated with TPF, trunk fat mass, android fat mass, gynoid fat mass, and android to gynoid ratio, while the inverse associations were observed in those with T2DM.

Prediabetes and T2DM are associated with the increased insulin resistance in target organs (e.g., liver, skeletal muscle, kidneys, brain, small intestine, and adipose tissue) and pancreatic β-cell dysfunction, with nearly 50% cell loss at the diagnosis of T2DM (1, 12). Although patients with prediabetes or T2DM are not necessarily obese, weight gain before DM develop is common (13). Obesity is recognized as the most powerful environmental risk factor among several modifiable risk factors for diabetes (14), which is associated with an increased insulin demand and increased likelihood of insulin resistance leading to prediabetes or hyperinsulinemia and ultimately T2DM (13, 15). Therefore, the results of our study that trunk fat mass, android fat mass, gynoid fat mass, android to gynoid ratio as well as TPF were higher in patients with prediabetic and T2DM than those without DM could be explained. Lee's study found that the predicted fat mass and percent fat estimated by anthropometric prediction equations were also positively associated with the risk of T2DM (16). A study of Japanese Americans found that greater visceral adiposity preceded the development of T2DM and also demonstrated an effect independent of fasting insulin, insulin secretion, glycemia, total and regional adiposity, and family history of diabetes (17). Furthermore, the investigation of associations between adiposity phenotypes and risk for incident prediabetes and diabetes of 732 obese adults found that visceral adiposity, increased liver fat, decreased lower body fat, insulin resistance, elevated triglycerides, and low adiponectin levels were associated with incident prediabetes and diabetes in obese individuals (18). The pathologic process of this increased insulin resistance may include the following aspects: the accumulation of excess fat leading to the increase of plasma free fatty acid (FFA) levels in obese patients may interfere with muscle insulin sensitivity and the increased FFA of those intra-abdominal tissues drained by portal circulation may lead to high FFA in portal vein, which may inhibit the hepatic clearance of portal insulin in turn. Besides, obesity may also cause the increased cortisol and androgen secretion leading to lower insulin sensitivity in muscle tissue and liver and physical and chronic psychologic stress may play an important role in exacerbating insulin resistance, prediabetes, and T2DM (11, 19).

The present study also showed that TPF, trunk fat mass, android fat mass, and gynoid fat mass decreased as the disease duration of T2DM increased, although it was of no significance for trunk fat mass and android fat mass. This may be because once an individual was diagnosed with T2DM, the use of anti-diabetic drugs, the intervention of diet, and exercise may cause weight loss and decrease in fat mass, and the elimination of blood sugar through urination at an extremely high sugar level may also make some contributions (20). A large amount of evidence showed that diet and exercise interventions can manage obesity and impair glucose regulation in T2DM (21), and it showed that every 1 kg loss in weight reduced the risk of diabetes by 16% (22). The oxidation of FFA during exercise was associated with insulin sensitivity, metabolic flexibility, and body fat mass (23, 24). As for drug therapy for patients with T2DM, metformin still remains the first-line therapy choice, for its excellent role in reducing hepatic glucose output, enhancing insulin sensitivity and lowering HbA1c by about 1–2% (4). There were studies showing that metformin can decrease food intake and body weight (25), with weight loss preferentially involving adipose tissue (26). Glucagon-like peptide-1 (GLP-1) receptor agonists such as liraglutide had been proved to sustain weight loss in obese patients and were associated with the reversal of prediabetes to normoglycemia during 1–2 years of follow-up (1) and another study showed that 3.0 mg of liraglutide was an adjunct to diet and exercise, associated with reduced body weight and improved metabolic control (27). In terms of insulin for the treatment of T2DM, a study by Haider showed that insulin or somatostatin infusion suppressed glucose-induced elevation of visfatin (a novel insulin-mimetic adipocytokine) (28), which can reduce fat accumulation and insulin resistance in patients with T2DM. All of these may coincide with the result of the decreased fat mass as the duration of T2DM increases in our study.

The blood glucose and HbA1c are considered as measures of DM control and parameters in relation to the risk of complications for decades. Of the two, HbA1c is more stable and convenient because of its absence of fasting (29). In this study, we found inverse associations of serum glucose and HbA1c with adiposity in patients with T2DM but direct correlations in those with prediabetes and without DM, which may be due to some drugs used for patients with T2DM being associated with weight gain in addition to their function of lowering blood glucose and HbA1c. In T2DM, there were 465 patients taking insulin and 1,436 patients taking glucose-lowering pills, with a few in prediabetes also doing so For example, sulfonylureas, such as gliclazide and glimepiride, were reported to have an association with hypoglycemia (30) and weight gain at the same time of their actions on β-cells to stimulate insulin secretion (31). Besides, thiazolidinediones, such as rosiglitazone and pioglitazone, were used clinically for improving of insulin sensitivity, might cause weight gain of up to 6 kg, which mainly because of fluid retention (4). Although insulin is an effective treatment to control blood glucose, weight and reduce HbA1c of 1.5–2% (4), it was also associated with a mean weight gain of 4 kg, especially in elderly patients (32). What's more, the bariatric surgery for those patients with T2DM and obesity was effective for weight reduction but also risky for hyperglycemia (33). Coincident with these findings, our study has implied that the simple measure of serum glucose, HbA1c or fat mass, and distribution may be insufficient to monitor the development and treatment effect of prediabetes and T2DM.

The main strengths of this study are the availability of a large, nationally representative population of US adults with data of fat mass and diabetes status from NHANES. Additionally, the availability of body composition measures by DXA offers additional information compared to the traditional measures of adiposity. More importantly, a large enough sample size allowed us to make the subgroup based on diabetes status and showed the distinct but neglected pattern of serum glucose and HbA1c with TPF and fat distribution that had never been reported in the previous studies.

This study also has several limitations. First, it is a cross-sectional study, which limits the inference of a causal correlation between serum glucose, HbA1c and TPF, fat distribution among adults. So, further basic mechanism research and large sample prospective study are still needed to identify the exact mechanism between them. Second, some NHANES participants were not eligible for a DXA scan because of excessive weight, height, or other reasons, so the estimates in this study might not fully represent the TPF and fat distribution in the general population. Third, there remains the possibility of bias caused by other potential confounding factors that we did not adjust for. Furthermore, some associations of disease duration, serum glucose, and HbA1c with adiposity in patients with T2DM were of no clinical or statistical significance, which may mainly because the number of patients with T2DM was small, so study on a larger sample of patients with T2DM is eagerly needed. Besides, TPF defined by DXA may have several limitations, so the body fat mass and distribution in prediabetes and T2DM requires in-depth research in multiple areas such as diagnostic criteria for obesity in T2DM patients using TPF measured by DXA, alteration in fat mass since prediabetes or T2DM is diagnosed and its detailed mechanisms.

## Conclusions

Our study indicated that adults with prediabetes and T2DM had significantly higher TPF, trunk fat mass, android fat mass, gynoid fat mass, and android to gynoid ratio compared with those without DM, and the fat mass decreased as the disease duration of T2DM increased. We also found inverse associations between serum glucose, HbA1c, and fat mass in patients with T2DM and direct association in those with prediabetes and without DM participants, which may give us a hint that just the measurements of serum glucose, HbA1c, or fat mass and distribution may be insufficient to monitor the development and treatment effect of T2DM.

## Data Availability Statement

The original contributions presented in the study are included in the article/supplementary material, further inquiries can be directed to the corresponding author/s.

## Ethics Statement

The Ethics Review Board of the National Center for Health Statistics approved all NHANES protocols.

## Author Contributions

JS and ZL contributed to data collection, statistical analysis, and writing and revising of the manuscript. ZZh and ZZe contributed to statistical analysis. WK supervised the study and contributed to polishing and reviewing of the manuscript. All authors contributed to the article and approved the submitted version.

## Funding

This work was supported by the CSCO-ROCHE Research Fund No. Y-2019 Roche-015, Beijing Xisike Clinical Oncology Research Foundation Y-HS2019-43, Wu Jieping Medical Foundation No. 320. 6750.19020 and 320.6750.2020-08-32, and CAMS Innovation Fund for Medical Sciences No. 2020-I2M-C&T-B-027.

## Conflict of Interest

The authors declare that the research was conducted in the absence of any commercial or financial relationships that could be construed as a potential conflict of interest.

## Publisher's Note

All claims expressed in this article are solely those of the authors and do not necessarily represent those of their affiliated organizations, or those of the publisher, the editors and the reviewers. Any product that may be evaluated in this article, or claim that may be made by its manufacturer, is not guaranteed or endorsed by the publisher.
